# Activatable MRI probes for the specific detection of bacteria

**DOI:** 10.1007/s00216-021-03710-z

**Published:** 2021-10-27

**Authors:** Prabu Periyathambi, Alien Balian, Zhangjun Hu, Daniel Padro, Luiza I. Hernandez, Kajsa Uvdal, Joao Duarte, Frank J. Hernandez

**Affiliations:** 1 Department of Physics, Chemistry and Biology, Linkӧping University, 58185 Linköping, Sweden; 2 Wallenberg Centre for Molecular Medicine, Linköping University, Linköping, Sweden; 3 Center for Cooperative Research in Biomaterials (CIC biomaGUNE), Basque Research and Technology Alliance (BRTA), 20014 Donostia-San Sebastián, Spain; 4 Department of Clinical and Experimental Medicine, Linkӧping University, Linköping, Sweden; 5 Department of Experimental Medical Science, Faculty of Medicine, Lund University, 22181 Lund, Sweden; 6 Wallenberg Center for Molecular Medicine, Lund University, Lund, Sweden

**Keywords:** MRI, Bacteria, Nucleic acid probes, Detection system, Nucleases, Activatable MRI contrast agents

## Abstract

Activatable fluorescent probes have been successfully used as molecular tools for biomedical research in the last decades. Fluorescent probes allow the detection of molecular events, providing an extraordinary platform for protein and cellular research. Nevertheless, most of the fluorescent probes reported are susceptible to interferences from endogenous fluorescence (background signal) and limited tissue penetration is expected. These drawbacks prevent the use of fluorescent tracers in the clinical setting. To overcome the limitation of fluorescent probes, we and others have developed activatable magnetic resonance probes. Herein, we report for the first time, an oligonucleotide-based probe with the capability to detect bacteria using magnetic resonance imaging (MRI). The activatable MRI probe consists of a specific oligonucleotide that targets micrococcal nuclease (MN), a nuclease derived from *Staphylococcus aureus*. The oligonucleotide is flanked by a superparamagnetic iron oxide nanoparticle (SPION) at one end, and by a dendron functionalized with several gadolinium complexes as enhancers, at the other end. Therefore, only upon recognition of the MRI probe by the specific bacteria is the probe activated and the MRI signal can be detected. This approach may be widely applied to detect bacterial infections or other human conditions with the potential to be translated into the clinic as an activatable contrast agent.

**Supplementary Information:**

The online version contains supplementary material available at 10.1007/s00216-021-03710-z.

## Introduction

All types of activatable probes share a unique feature, they are turned “on” only after encountering the specific target or physical stimulus, while they remain “off” in the absence of such events [1, 2]. Activatable probes have shown remarkable sensitivity and specificity because their target signal is maximized and the background signal is suppressed, facilitating a higher target-to-background ratio [3, 4]. Activatable probes can detect biological events at the nanoscale level (e.g., conformational changes or molecular recognition) using transduction mechanisms such as Förster resonance energy transfer (FRET) [5–8] and surface plasmon resonance (SPR) [9]. The best example in this regard is FRET where, upon excitement, a donor chromophore transfers its energy to an acceptor chromophore through non-radiative dipolar coupling. This energy transfer event is governed by one factor of the donor–acceptor distance [10]. Based on distance-dependent properties, activatable fluorescent probes allow the measurement of intracellular events, providing a powerful tool for studying fundamental aspects of cell biology and molecular recognition [11]. However, a major setback with this technology is the interference observed during the light–matter interactions, especially while dealing with in vivo biological systems like opaque tissues or organs embedded deep within the body [12]. Interestingly, some approaches based on enzymatic or small-molecule probes have been reported for the identification of bacteria using fluorescence tracers [13, 14]. In the last decades, activatable MRI contrast agents have been described [15–18], providing the opportunity to detect biochemical events at the cellular and molecular levels using MRI. We and others have previously shown a quenching effect on activatable MRI probes under different experimental conditions. Santra et al. [19] have encapsulated SPION and Gd^3+^ using pH polymeric materials, allowing the non-specific activation of the MRI probes by pH changes. We have then suggested that the distance between SPION and Gd^3+^ is a key parameter for constructing effective activatable MRI probes, using nucleic acids as linkers [20]. Much later, Choi et al. have confirmed our initial findings by measuring the critical distance needed to obtain activatable MRI probes [21, 22]. Activatable MRI probes can overcome the limitation of fluorescence-based systems, with a mature MRI technology that offers the advantages of unlimited tissue penetration and high spatial resolution, which are exceptional features for clinical approaches [23].

In this study, we developed an activatable MRI probe with a “turn-on” switch for detecting a specific nuclease, micrococcal nuclease (MN) secreted by *Staphylococcu*s *aureus* (*S. aureus*). *S. aureus* is a major cause of bacterial infections in humans and its virulence is characterized by high morbidity and mortality, as in the notorious case of methicillin-resistant *S. aureus* (MRSA) infections [24]. More generally, *S. aureus* is behind severe and difficult to diagnose conditions that may implicate resistant or non-resistant strains, such as osteomyelitis, endocarditis, infantile pneumonia, or septic arthritis, with significant clinical and economic impact [25]. For these reasons, the prevention and control of *S. aureus* has been identified as a public health priority. In order to prevent and control *S. aureus*-related infections, rapid and accurate diagnostic methods are essential for the timely and appropriate treatment, especially in the case of MRSA.

**Fig. 1:**
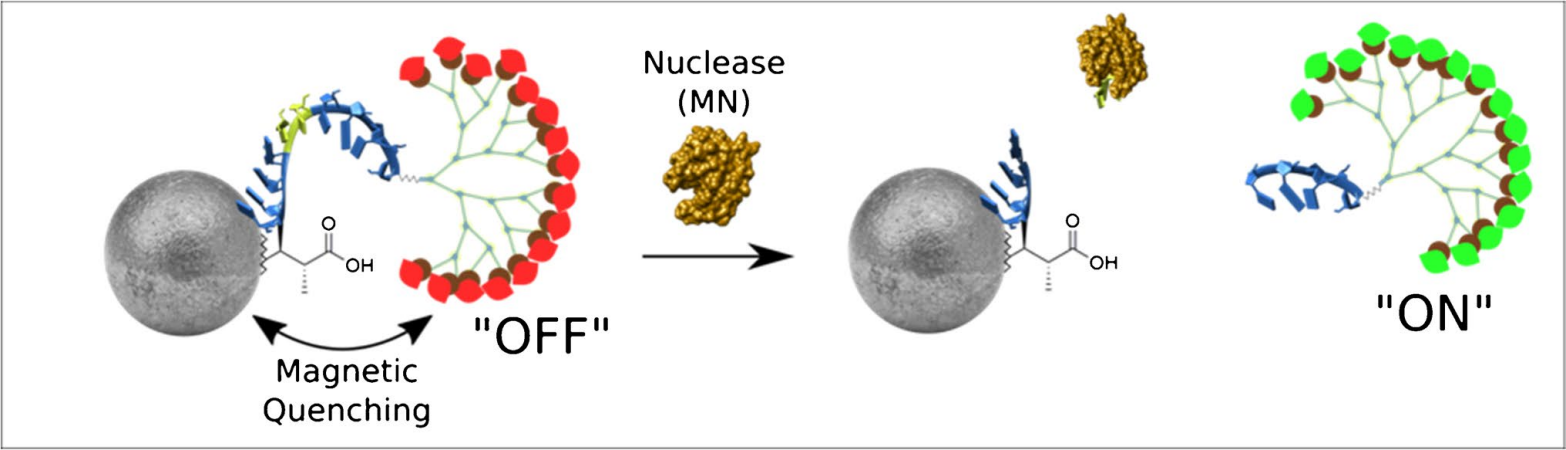
Activation of the MRI probe by specific nuclease activity derived from bacteria

In our study, we use nucleases as biomarkers. Nucleases are proteins with tremendous diversity and widespread expression that could be used to specifically identify a variety of bacterial species [26]. Besides their detection by immune assays or by molecular techniques, as degrading enzymes, nucleases can be detected by their activity towards/degradability of oligonucleotide substrates. This activity can be therefore detected by activatable oligonucleotide probes that can be further incorporated in different types of diagnostic biosensors. Herein, we report on the development of activatable MRI probes that detect bacteria and work as a contrast agent. In a previous proof-of-concept study, a molecular imaging approach that rapidly and specifically detects *S. aureus* infections was reported. This activatable fluorescent probe, the sequence of which is referred to as TT probe, is turned “on” by micrococcal nuclease (MN) specifically secreted by these bacteria [27]. With this strategy, we have developed an oligonucleotide probe with high sensitivity and specificity that allow in vivo detection of bacteria in 45 min, which clearly demonstrates the potential of this technology for targeting applications where nuclease activity could be detected. We have previously demonstrated the specificity of TT probe to MN secreting *S. aureus* among other strains and other bacteria in addition to the resistance of the probe in human and mouse serum [27]. Based on this knowledge and as a proof of concept study, we have designed an activatable MRI probe composed of a core superparamagnetic iron oxide nanoparticle (SPION) bearing *S. aureus*–specific short oligonucleotide sequences that are linked to dendrons functionalized with several gadolinium ion (Gd^3+^) complexes. Incorporation of dendrons in MRI contrast agents and the resulted benefits such as the enhanced relaxivity have been widely described [28–31]. In the MRI probe described in this paper, the short distance between SPION and Gd^3+^ induces a quenching effect of SPION to the magnetic relaxation (*T*_1_-based) of Gd^3+^, and this effect is described as “magnetic quenching.” Then, in the presence of the nucleases associated to *S. aureus* bacteria, the oligonucleotides are specifically cleaved, thus releasing Gd^3+^ complexes from the superparamagnetic SPION core, resulting in “on” *T*_1_ relaxation. Figure 1 depicts the schematic structure and mechanism of activatable MRI probes for detecting specific bacteria using nucleases as biomarkers and oligonucleotides as recognition molecules. To the best of our knowledge, this is the first approach that detects bacteria-derived protein with an activatable MRI probe using SPIONs and dendrons functionalized with Gd^3+^. This work reports an efficient activatable MRI probe that exhibits high sensitivity due to high signal-to-background ratios and with unlimited tissue penetration provided by the MRI signal acquisition; these features facilitate the translation of this approach into the clinical settings.

## Material and methods

### Materials

Oleic acid-coated iron oxide nanoparticles (20-nm SPION) dispersed in chloroform were obtained from Ocean NanoTech (San Diego, CA). GdCl_3_, meso-2,3-dimercaptosuccinic acid (DMSA), tryptic soy agar (TSA), and tryptic soy broth (TSB) were purchased from Sigma-Aldrich (St. Louis, MO, USA). Sulfosuccinimidyl 4-(N-maleimidomethyl) cyclohexane-1-carboxylate (Sulfo-SMCC), sodium L-ascorbate, and copper (II) sulfate were purchased from Thermo Scientific, Uppsala, Sweden. bis-MPA ammonium dendron, acetylene core, Generation 4 (G4) were obtained from Polymer Factory, Sweden AB. *p*-SCN − Bn − DTPA was obtained from Macrocyclics, USA. Tris [3-hydroxypropyltriazolylmethyl] amine (THPTA) was purchased from Click Chemistry Tools LLC. PD MiniTrapTM G-10 was purchased from GE Healthcare. MiniMACS separator and MACS MS column were purchased from Miltenyi Biotec (cat#130–042-102). All other chemicals and reagents were of reagent grade and used without further purification.

### Methods

#### Preparation of the DMSA-coated SPION (D-SPION)

Ligand exchange was performed to exchange capping molecule from oleic acid (organic phase) to DMSA (water-soluble phase) on surface of 20-nm iron oxide nanoparticles (SPION). Two milligrams of 20-nm SPION in 500 µL of toluene and DMSA [5 mg] in 500 µL of methanol were mixed and shaken (1000 rpm) for 12 h at room temperature (RT) until two phases appear. After incubation, the nanoparticles were centrifuged at 3000 rpm for 10 min. The final black solid was air dried and redispersed in distilled water. The pH of the resulted solution was adjusted to 10. The unreacted DMSA was removed via MACS MS column.

#### Labelling of TT probe on D-SPION (D-SPION-TT-N_3_)

A total of 150 µL of Sulfo-SMCC (5 mM) was dissolved into 100 nmol (10 µL) of TT probe (5′-NH_2_-mC-mU-mC-mG-**T-T**-mC-mG-mU-mU-mC-N_3_-3′) and the mixture was shaken at 1500 rpm for 1 h at RT. Next, 0.25 mL of D-SPION was added and the mixture was shaken at 500 rpm for an additional 2 h at RT [32]. D-SPION@TT-N_3_ probe was then transferred to 500 μL of sodium bicarbonate buffer pH 8.8 using a MACS MS column.

#### ***Click chemistry CuAAC reaction (D-SPION@TT-G***_***4***_***)***

To carry out the reaction, first, 500 µL of D-SPION@TT-N_3_ was mixed with 100 µL of G4 dendron alkyne (0.06 mmol) prepared in DMF. Next, 50 μL of 500 mM THPTA/100 mM CuSO_4_ and 25 μL of sodium l-ascorbate (50 μmol) were added to the reaction mixture. After 3 h of incubation, the nanoparticles were purified on a MACS MS column with sodium bicarbonate buffer, pH 8.8. Then, the D-SPION@TT-G_4_ probe was transferred to 500 μL of sodium bicarbonate buffer at pH 8.8 by MACS MS column [33].

#### Preparation of the activatable MRI probe

Finally, D-SPION@TT-G_4_ was first reacted with DTPA followed by Gd^3+^ chelation using gadolinium chloride. The reaction was performed as follows: 300 μL of D-SPION@ TT-G_4_ was mixed with 500 μL of *p*-SCN-DTPA (5 mg; sodium bicarbonate buffer pH 8.8) at RT, overnight. The pH of the reaction mixture was adjusted to 8 ~ 9 by adding 1 N NaOH and MACS MS column was used to purify D-SPION@TT-G_4_-DTPA NPs from the mixture. The D-SPION@TT-G_4_-DTPA was transferred to water (pH adjusted to 7.4) to a final volume of 500 μL. In total, 200 μL GdCl_3_ solution (2.64 mg) was added to 300 μL of D-SPION@TT-G_4_-DTPA solution at RT. The solution was adjusted to pH 8 and stirred overnight. The resulting probe (D-SPION@TT-G_4_-Gd) was purified with a MACS MS column to a final volume of 250 μL aqueous solution.

#### Activatable MRI probe characterization

##### Transmission electron microscopy (TEM)

The size and shape of the activatable MRI probe were analyzed using TEM (with a FEI Tecnai G2 operated at 200 kV). A total of 10 μL of probe dispersed in an aqueous solution (2 mg mL^−1^) was deposited directly on an amorphous carbon film supported copper TEM grid.

##### Dynamic light scattering (DLS)

The hydrodynamic diameters of D-SPION and MRI activatable probe were measured using a Zetasizer Nano ZS90 (Malvern Instruments Ltd., Worcestershire, UK). Dispersion D-SPION and activatable MRI probe were prepared at a concentration of 0.1 mg mL^−1^ in deionized water, ultrasonicated for 60 min. Next, the solution was filtered with a 0.22-µm filter and finally subjected to analysis.

##### UV–Vis spectroscopy

PerkinElmer Lambda 950 UV–Vis spectrometer was used to record the absorbance of D-SPION, TT oligonucleotide probe, and the activatable MRI probe. The samples were diluted in deionized water (0.1 mg mL^−1^) and the spectra were recorded at the wavelength of 200–800 nm.

##### Magnetic quenching evaluation

Preparation of G4-dendron-Gd^3+^ complex was obtained by conjugating a solution of G4-dendron- NH_2_ (1.0 mg, 0.043 mmol) with p-SCN-Bn DTPA (5 mg, 0.39 mmol) in 1 mL (0.1 M sodium bicarbonate buffer) at RT. The solution was stirred for 12 h, then filtered 3 times using an Amicon Ultra-15 (3000 MWCO) column at 4500* g* for 5 min. G_4_-Dendron-DTPA (10, 20, 30, 40, and 50 µg) was added to different concentrations of 0.04, 0.08, 0.12, 0.16, and 0.2 mM GdCl_3_·6H_2_O in 220 µL for 2 h at 40 °C, respectively. Then, all samples were loaded in 7.5-mm-diameter NMR tubes and analyzed with a Bruker minispec mq60 NMR analyzer at 37 °C using a magnetic field of 1.41 T (inversion time (IT) = 50, 100, 200, 300, 500, 1000, 2000, and 3000 ms). Each sample was temperature-stabilized for 4 min before measurement. Then, *r*_1_ values calculated from the slope of 1/*T*_1_ graph were plotted against the Gd^3+^ concentration and compared with the activatable MRI probe under the same conditions.

##### Inductively coupled plasma mass spectroscopy

The quantification of Fe and Gd was performed using ICP-MS by ALS Scandinavia AB (Sweden).

#### Preparation of bacterial supernatant

*Staphylococcus aureus* (ATCC 29,213) and *Staphylococcus epidermidis* (ATCC 39,584) were cultivated in respective petri dishes with tryptic soy agar (TSA) medium supplemented with defibrinated sheep blood (Thermo Scientific, Inc., Waltham, MA, USA). Growth of individual bacterial colonies was allowed using the quadrant method by streaking a porous glass bead directly in the petri dishes. Following 24-h incubation at 37 °C, a single colony was transferred from solid media into 50 mL tryptic soy broth (TSB) and incubated for 24 h at 37 °C and 200 rpm. The liquid cultures were diluted (1:500) in TSB and incubated for 24 h at 37 °C and 200 rpm. The bacterial culture is estimated to be in the stationary phase with values greater than 10^9^ CFU/mL after this incubation time of 24 h. Next, the culture was centrifuged at 4500 × *g* for 30 min and supernatants were collected for direct use or storage at 4 °C or − 20 °C. TSB culture medium was used as control [34]. Bacterial counts were calculated using McFarland standards and reported as colony forming units (CFU) per milliliter.

#### *** Relaxation T***_***1***_*** measurement using NMR Minispec (1.41 T)***

The activatable MRI probes at different Fe concentrations (0.005, 0.01, 0.02, 0.03, and 0.04 mM) were incubated with *S. aureus* (targeting bacteria) and *S. epidermidis* (non-targeting bacteria) cultivated media or TSB. All reaction mixtures were incubated at 37 °C for 1 h and loaded into 7.5-mm-diameter NMR tubes and measured with a Bruker minispec mq60 NMR analyzer at 37 °C using a magnetic field of 1.41 T (IT) = 50, 100, 200, 300, 500, 1000, 2000, and 3000 ms. Each sample was temperature stabilized for 4 min before measurement. Then, *r*_1_ values calculated from the slope of the 1/*T*_1_ graph were plotted against the Fe concentration.

#### MR imaging measurements

The MRI nanoprobe was incubated for 1 h with culture media, *S. epidermidis* (non-targeting bacteria) and *S. aureus* (targeting bacteria). Then, the probe was loaded into Wilmad NMR tubes 3-mm diameter at concentrations of 0.03 and 0.07 mM Fe and incubated at 37 °C during MRI scan. MRI scans were carried out in a preclinical 9.4-T magnet (Agilent, Palo Alto, CA, USA) interfaced to Avance III electronics, using a quadrature transmit-receive coil (Bruker, Ettlingen, Germany). *T*_1_ values were estimated from images acquired using the rapid acquisition with relaxation enhancement (RARE) sequence with inversion recovery (IT = 50, 200, 400, 800, 1500, 3000, 5500, 8000, 12,000 ms, TE = 7.0 ms, echo train length 2, data matrix size 128 × 64, field of view 30 × 15 mm^2^, slice thickness = 3 mm, 1 scan).

#### Cell culture and cytotoxicity assay

The CCD18LU fibroblasts obtained from American Type Culture Collection (ATCC) (Manassas, VA, USA) were seeded in 96-well clear bottom plates, at the density of 1 × 10^4^ cells/well. The cells were maintained in minimal essential medium (MEM) supplemented with fetal bovine serum (FBS), l-glutamine, and penicillin/streptomycin. All cell culture reagents were purchased from Gibco (Thermo Fisher Scientific, USA). After 24-h incubation at 37 °C, the cells were treated for 24 or 48 h with 100µL cell culture medium containing MRI probe at 0.07 mM Fe. Untreated cells were supplemented with fresh medium only. Next, nucleic acid stain (CyQUANT Direct Cell Proliferation Assay Kit, Invitrogen, UK) was added at a volume of 100µL/well. After 1-h incubation at 37 °C, bright field and fluorescence microscopy images were acquired using Cytation 1 (Cell Imaging Multi-Mode Reader, BioTek, USA), and Gen5 software with a × 4 optical objective was used for cell viability counts.

## Results

**Fig. 2:**
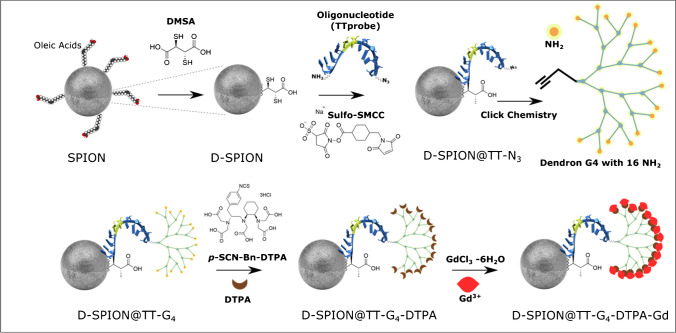
Schematic of the activatable MRI probe synthesis

We and others have reported that *T*_1_ relaxivity of Gd^3+^ can be quenched by superparamagnetic (SPION) in the local magnetic field [35]. The high transverse relaxivity (*r*_2_) effect of SPION serves as a quencher to control the longitudinal relaxivity (*r*_1_), and practically negligible *T*_1_ contrast effects for activatable MRI probes are observed [33]. Figure 2 shows the schematic synthesis route of the activatable MRI probes used in this study. The synthesis begins with SPIONs of 20-nm diameter that were modified with dimercaptosuccinic acid (DMSA). This step converts the hydrophilic nanoparticles into hydrophilic SPIONs with coupling groups for further conjugation (D-SPION). An oligonucleotide (TT probe) that specifically recognizes nucleases derived from *S. aureus* was used as a biorecognition molecule [36].

The oligonucleotide TT probe was synthesized with amine and azide coupling groups at 5′ and 3′-end, respectively, for further conjugation. Finally, the activatable MRI probe was assembled by flanking the oligonucleotide TT probe with D-SPION at the 5′-end and by the dendron functionalized with gadolinium complex at the 3′-end. A detailed description of the synthesis route is provided in the “Methods” subsection. To confirm the amounts of Fe and Gd^3+^ bared by the activatable MRI probes, we quantified the elemental composition by inductively coupled plasma mass spectroscopy (ICP-MS) analysis. The ICP-MS analysis determined concentrations of 8, 23, and 270 mg/kg for Fe and Gd^3+^, respectively. These results suggest an efficient functionalization of Gd^3+^ complexes over the D-SPION surface, and a successful methodology to obtain activatable MRI probes.

**Fig. 3:**
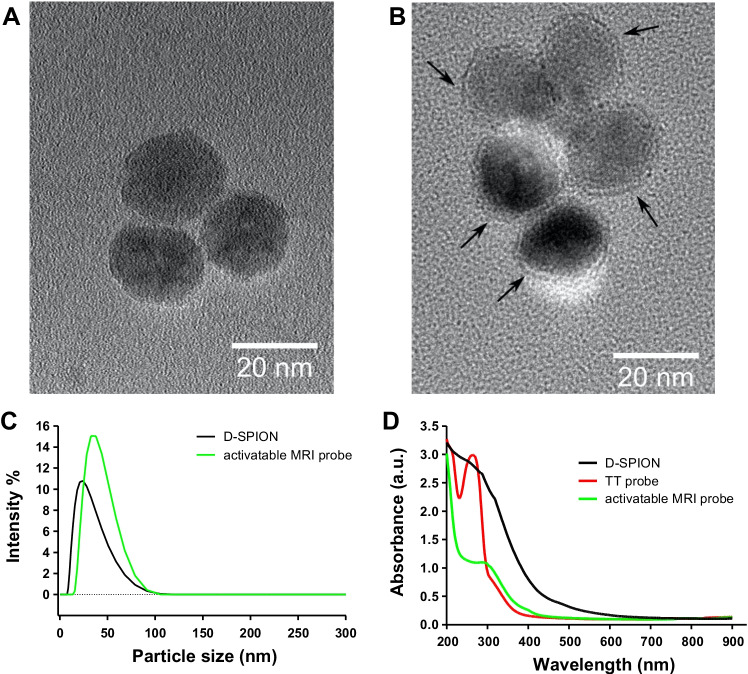
Characterization of activatable MRI probes. TEM images were acquired for (A) D-SPION and (B) activatable MRI probe; the arrows show the solvation layer between D-SPION and Gd^3+^. (C) The hydrodynamic sizes of D-SPION and activatable MRI probe were obtained by DLS analysis. (D) The UV–Vis profiles of D-SPION, oligonucleotide TT probe, and activatable MRI probe were obtained by UV–Vis spectroscopy analysis

Next, microscopy features such as size and monodispersity of D-SPIONs and activatable MRI probes were evaluated by transmission electron microscopy (TEM). Figure 3A shows D-SPION hydrophilic nanoparticles with an ellipsoidal shape with an approximate size of 20 nm. Interestingly, Fig. 3B shows an increase in size, up to 24 nm, for the activatable MRI probes. This ~ 4-nm increase in size is consistent with the oligonucleotide and dendron conjugation that can be observed as an interstitial space (Fig. 3B, arrows) between the D-SPION and the solvation layer decorated with Gd^3+^. These results are in good agreement with previous TEM images of similar MRI probe constructs [37]. Next, the colloidal stability in water of the D-SPION and activatable MRI probes has been evaluated by DLS. Figure 3C shows the mean hydrodynamic size of 23.26 nm for D-SPION, which is slightly larger when compared with the 20 nm reported by TEM analysis. The activatable MRI probe construct has reported an average hydrodynamic size of 34.85 nm. These results are consistent with the conjugation of oligonucleotide and dendron thickness that form the solvation layer observed by TEM (Fig. 3B). Altogether, TEM and DLS results confirmed that no aggregation occurs after the synthesis of the activatable MRI probes and hydrodynamic size distributions suggest a monodisperse colloidal system. To complete the characterization of the activatable MRI probes, UV–Vis absorption spectroscopy was performed to confirm the incorporation of the oligonucleotide during the synthesis process. These measurements were carried out in water at room temperature. Figure 3D shows the individual UV–Vis profile of D-SPION (black line), where no clear peak was observed. In contrast, the UV–Vis profile of the oligonucleotide (TT probe) has shown a clear peak at 264 nm that is consistent with the chemically modified oligonucleotides (red line). Furthermore, the activatable MRI probe profile (green line) has exhibited a broad peak from 285 to 340 nm indicating the presence of the oligonucleotide in the MRI construct. These results confirm the successful synthesis of the activatable MRI probes, and more specifically the incorporation of the oligonucleotide into the MRI probes.

**Fig. 4:**
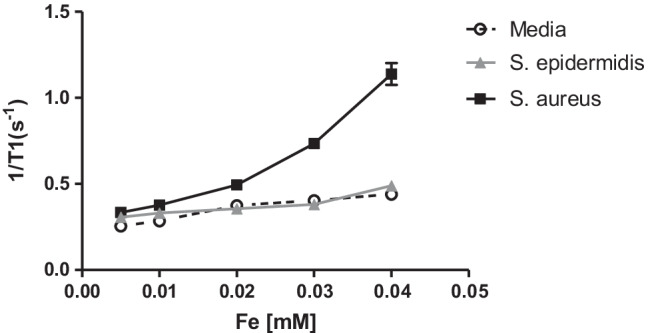
Activatable MRI probe performance. (A) Schematic mechanism of action of the activatable MRI probes. (B) Specific detection of bacteria using activatable MRI probes and data acquisition by Minispec 1.4 T

Once the activatable MRI probes were synthesized and fully characterized, we tested their functionality as contrast agents. To demonstrate the functionality of the activatable MRI probes, first we calculated the “magnetic quenching” obtained after synthesis. We therefore compared the *T*_1_ relaxivity signals of dendron G4 functionalized with Gd^3+^ (Dendron-Gd^3+^ complex) and the activatable MRI probes. Dendron-Gd^3+^ complexes (Fig S1, squares) have reported higher *T*_1_ relaxivity signal of 46.018 mM^−1^ s^−1^ (r_1_), led by prolonged rotational correlation time (τR). In contrast, the activatable MRI probe with the Gd^3+^ quenched by the close proximity with D-SPION (Fig S1, circles) has shown a lower *T*_1_ relaxivity signal of 12.586 mM^−1^ s^−1^, resulting in a *T*_1_ signal difference of 3.66-fold. These results indicate that the “magnetic quenching” effect was obtained after synthesis and that the activatable MRI probes are ready to be tested using biological samples. Furthermore, we have tested the cytotoxicity of the MRI probe by a fluorescence-based cell viability assay. The results of cell viability counts have shown that cells were not significantly affected after incubating the MRI probe (0.07 mM Fe) for 24 and 48 h (Fig S2). Next, we sought to evaluate whether the activatable MRI probes have the capability to identify bacteria in a specific manner, in standard bacterial cultures after 24 h. Thus, the activatable MRI probes were incubated for 1 h at 37 °C with bacterial cultures of *S. aureus* as the target bacteria (2.4 × 10^9^ CFU/mL) and *S. epidermidis* as the control bacteria (2.1 × 10^9^ CFU/mL), along with bacterial culture media as background control. Importantly, *S. epidermidis* does not produce MN, the nuclease needed to activate the MRI probes. Then, the *T*_1_ relaxivities of Gd^3+^ derived from the MRI probes were measured at 60 MHz using an NMR MiniSpec (Fig. 4). The MiniSpec device allows the acquisition of rapid and simple relaxivity measurements that facilitate the optimization of the activatable MRI probes. The relaxivity results have shown that *S. epidermidis* and bacterial culture media did not show a significant change at several concentrations of the activable MRI probes. In contrast, a clear shift was observed when the MRI probes were incubated with *S. aureus*, showing higher relaxivity signals than both controls. Moreover, a concentration-dependent increase in relaxivity signal was only observed for *S. aureus* samples. These results confirm the specific recognition of a bacterium using an activatable MRI probe and the capability of this approach to add diagnostic value to the MRI-based systems by incorporating a novel type of contrast agent.

**Fig. 5:**
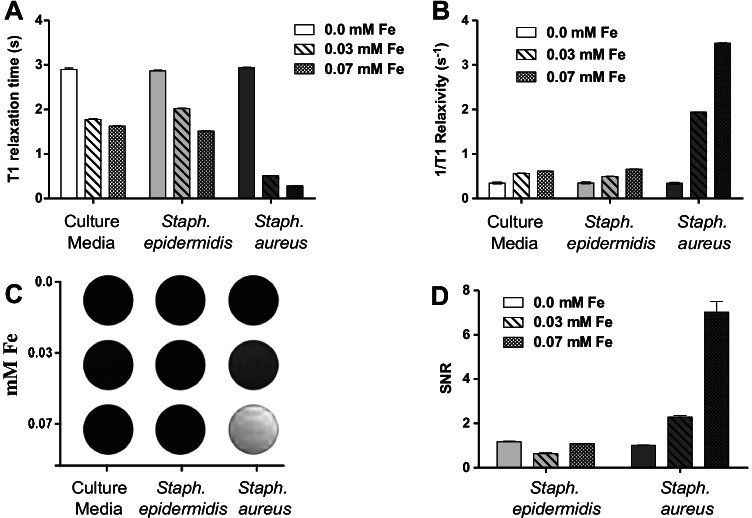
Specific detection of bacteria using 9.4-T MR imaging. (A) *T*_1_ relaxation times for *S. aureus* (target bacteria), and controls: *S. epidermidis* (non-target bacteria) and culture media. (B) Bar graph of *R*_1_ relaxation rate values for target bacteria and controls. (C) Contrast changes in *T*_1_-weighted MR phantom imaging reflecting the activation of the MRI probe*.* (D) SNR values for *S. aureus* and *S. epidermidis.* Data are shown as mean ± SD of *n* = 3 experiments

To demonstrate the potential applicability in the clinic of this type of activatable MRI probes, we have repeated the detection of specific bacteria using an MRI machine (Bruker BioSpec Avance III). For the MRI analysis, we used the same samples and experimental conditions described in the previous relaxivity studies. Figure 5A shows the *T*_1_ relaxation times acquired by the MRI machine. The samples containing *S. aureus* have shown lower relaxation times compared with both controls, non-targeted bacteria and culture media, suggesting that this activatable strategy is an effective manner of identifying bacteria using an MRI system. Furthermore, we calculated the relaxation rate 1 (*R*_1_) as the inverse of relaxation time (1/*T*_1_ relaxivity S^−1^). Figure 5B shows the *R*_1_ calculations, and any value over 1 (arbitrary threshold) is considered positive for *S. aureus*. Consistent with the previous results, the MR images acquired with an 9.4-T magnet have shown an increase in intensity for Gd^3+^ when the activatable MRI probes were incubated with *S. aureus* samples, whereas no change in intensity was observed for *S. epidermidis* and bacterial culture media (Fig. 5C). Moreover, a concentration-dependent effect in brightening from lower to higher concentrations of activatable MRI probes (0, 0.03, and 0.07 Fe mM) was also observed for *S. aureus* samples. Next, we calculated the signal-to-noise ratios (SNR) using the culture media as control (Fig. 5D). We have found 3.80- and 6.49-fold signal increases for *S. aureus* samples over *S. epidermidis* (non-targeted bacteria) when the activatable MRI probe was used at 0.03 and 0.07 mM concentrations (Fe), respectively. In contrast, *T*_2_ relaxation times, the *r*_2_ calculations, the MR images, and the *T*_2_ SNR have shown limited capability to differentiate *S. aureus* from *S. epidermidis* (Fig S3). In addition, the *T*_2_ SNR calculations have reported 1.47- and 1.70-fold signal increases for *S. aureus* samples over *S. epidermidis* using 0.03 and 0.07 mM concentrations (Fe), respectively. The comparative analysis of the *T*_1_ and *T*_2_ signals demonstrates that the activatable MRI probes reported in this study behave more efficiently as a *T*_1_ rather than a *T*_2_ contrast agent. All together, these results confirm the feasibility of activatable MRI probes for detecting biological markers such as nucleases derived from bacteria, demonstrating the potential of this approach for in vivo translation.

## Discussion

The development of activatable MRI probes that respond to a biochemical stimulus has increased the attention of the scientific community in the last decade. Specific detection of biomarkers such as sugars, proteins, and enzymes has expended the diagnostic capabilities of MRI technologies by providing information at the cellular and molecular levels. These emerging activatable features, combined with the anatomical and functional imaging of standard MRI systems, can create alternative diagnostic strategies beyond the currently existing methods in the clinic.

This study reports, for the first time, on an activatable contrast agent with the capability to detect bacteria in a specific manner using MRI. The diagnosis of infectious diseases such as osteomyelitis, where *S. aureus* is one of the most prevalent pathogens, can be significantly improved by combining the specificity provided by our MRI probe and the unlimited tissue penetration features of the MRI technology. We envision that this approach could be potentially expanded to other bacteria or other human conditions, such as cancer.

## Supplementary Information

Below is the link to the electronic supplementary material.

Supplementary file1 (DOCX 10445 KB)
