# Supplementary material for: Activatable MRI probes for the specific detection of bacteria
Source: Anal Bioanal Chem. 2021 Oct 27;413(30):7353–62. doi: 10.1007/s00216-021-03710-z (PMC8626403; doi:10.1007/s00216-021-03710-z)
Supplement: Supplementary file 1 — Supplementary file1 (DOCX 10445 KB) [file 216_2021_3710_MOESM1_ESM.docx]

**Activatable MRI probes for the specific detection of bacteria**

Prabu Periyathambi^1,2^, Alien Balian^1,2^, Zhangjun Hu^1^, Daniel Padro^3^, Luiza I. Hernandez^4^, Kajsa Uvdal^1^, Joao Duarte^5,6^ and Frank J. Hernandez*^1,2^

^1^ Department of Physics, Chemistry and Biology, Linkӧping University, Linköping, 58185, Sweden

^2^ Wallenberg Centre for Molecular Medicine, Linköping University, Linköping, Sweden

^3^ CIC biomaGUNE, San Sebastian, 20014, Spain

^4^ Department of Clinical and Experimetal Medicine, Linkӧping University, Linköping, Sweden

^5^ Department of Experimental Medical Science, Faculty of Medicine, Lund University, Lund, 22181, Sweden

^6^ Wallenberg Center for Molecular Medicine, Lund University, Lund, Sweden

* To whom correspondence should be addressed. frank.hernandez@liu.se

**Supplementary information**

**Figure S1.** Magnetic quenching effect. *T_1_* measurement of dendon-Gd^3+^ complex and activatable MRI probes were performed to demonstrate the magnetic quenching effect on the MRI probes.

**Figure S2.** Cytotoxicity assessment of the MRI-probe. A. Viability of fibroblast cells was determined when treated with the MRI-probe (0.07 mM Fe) for 24h, along with untreated cell as control. B. Viability assessment after 48h. Viability cell counts were performed at 24h (C), and after 48h (D).

**Figure S3.** Specific detection of bacteria using 9.4T MR imaging. A) *T_2_* relaxation times for *S. aureus* (target bacteria), and controls: *S. epidermidis* (non-target bacteria) and culture media. B) Bar graph of *R_2_* (1/T2) relaxivity rate values for target bacteria and controls. C) Contrast changes in *T_2_*-weighted MR phantom imaging reﬂecting the activation of the MRI probe. D) SNR values for S. aureus and S. epidermidis. Data are mean±SD of n=3 experiments.
